# Case Report: Identification of Maternal Low-Level Mosaicism in the Dystrophin Gene by Droplet Digital Polymerase Chain Reaction

**DOI:** 10.3389/fgene.2021.686993

**Published:** 2021-07-01

**Authors:** Pengzhen Jin, Xiaoyang Gao, Miaomiao Wang, Yeqing Qian, Jingjin Yang, Yanmei Yang, Yuqing Xu, Yanfei Xu, Minyue Dong

**Affiliations:** ^1^Women's Hospital, School of Medicine Zhejiang University, Hangzhou, China; ^2^Key Laboratory of Reproductive Genetics, Ministry of Education, Zhejiang University, Hangzhou, China

**Keywords:** Duchenne muscular dystrophy, germline mosaicism, droplet digital polymerase chain reaction, gap-polymerase chain reaction, *de novo* mutations

## Abstract

Germline mosaicism should be suspected when the same de novo mutations are identified in a second pregnancy with asymptomatic parents. Our study aims to find a feasible approach to reveal the existence of germline mosaicism. Multiplex Ligation-dependent Probe Amplification was performed on a Duchenne muscular dystrophy affected pedigree to detect deletion mutations. Then gap-polymerase chain reaction was performed to amplify the breakpoints junction sequence. Droplet digital polymerase chain reaction was utilized to identify the mutation frequencies in healthy parents. The same deletion in the exon 51 of the dystrophin gene, which was 50,035 bp in size, was detected in the proband and the fetus but not in their parents. Droplet digital polymerase chain reaction analysis of peripheral blood samples revealed mutant alleles of 3.53% in maternal blood cells. We here report a case of maternal low-level mosaicism confirmed by droplet digital polymerase chain reaction in peripheral blood samples, which reveals the existence of germline mosaicism. Gap-polymerase chain reaction combined with droplet digital polymerase chain reaction provide insights into the detection of germline mosaicism.

## Introduction

*De novo* mutations (DNMs) refer to genetic changes in the offspring that cannot be detected in the genome of either parent (Haldane, 2004; Wilfert et al., 2017). The recurrent risks for DNMs are theoretically low, even non-existent. However, cautions should be taken when the same DNMs repeatedly occur in two or more offspring: germline mosaicism might be the possible explanation of it (Bakker et al., 1987; Campbell et al., 2014; Di Donato et al., 2014; Patel et al., 2018; Qian et al., 2019).

The term mosaicism is used when two or more genetically different cell populations present in an individual (Patel et al., 2018; Jin et al., 2020). The incidences of somatic mosaicism are estimated to be notably higher, comparing with germline mosaicism, ~512-fold to 3,312-fold (Campbell et al., 2014; Milholland et al., 2017). In addition, germline mosaicism is theoretically accompanied by low-level somatic mosaicism. The evidences above indicate that the identification of low-level somatic mutations is an efficient way to reveal the existence of germline mosaicism (Patel et al., 2018).

Droplet digital PCR (ddPCR) is an advanced technology developed based on the traditional digital PCR. The samples are randomly divided into tens of thousands of oil droplets and each droplet is analyzed individually (Hindson et al., 2011, 2013; Pinheiro et al., 2012). It has been reported that mutations can be quantitatively detected by ddPCR with a sensitivity below 0.001% (Postel et al., 2018). The application of ddPCR makes it a reality to identify low-level mosaicism.

In this study, we reported two consecutive conceptions with the same 50 kb deletion in the exon 51 of the dystrophin gene. It is demonstrated that the mutant frequency in the mother is 3.53%, a low-level mosaicism which could not be identified by Multiplex Ligation-dependent Probe Amplification (MLPA). The application of ddPCR to identify low-level mosaicism has provided insights into germline mosaicism.

## Materials and Methods

### Case Report

The proband, male, 10 years old, was the first child of the healthy non-consanguineous couple. The child was born at term and the process of pregnancy and delivery was normal. Duchenne muscular dystrophy (DMD) was diagnosed at the age of 28 months. The enzyme profile was as following: serum creatine kinase (CK), 15,413 IU/L (ref: 25–200 IU/L) and creatine kinase isoenzyme (CK-MB), 527 IU/L (ref: <25 IU/L). The electromyography revealed a mild myogenic damage. MLPA analysis revealed a deletion of the exon 51 of the dystrophin gene. Based on the clinical examination, the diagnosis of DMD was established. MLPA was performed on the asymptomatic couple and no deletions were detected. The couple came to our hospital for prenatal diagnosis because the 32-year-old mother was at 20 weeks of gestation.

The study was approved by the Ethics Committee of Women's Hospital, School of Medicine Zhejiang University (IRB-20200129-R). All participants were provided a written informed consent.

### Amniocentesis and Fetal Karyotyping

Transabdominal amniocentesis was performed under real-time sonographic guidance. A total of 20 ml of amniotic fluid was aspirated after discarding the first 2 ml of amniotic fluid. Amniotic fluid cells were cultured and chromosome karyotype analysis was conducted on metaphase preparations with targeted 400-band level. Generally, 30 metaphases were counted and it was extended to 50 metaphases if different cell lineages were identified in the same patient. Microscopic karyotype was conducted using the GSL-120 CytoVision platform (Leica, German). Chromosomal karyotype was described according to the International System for Human Cytogenetic Nomenclature (Stevens-Kroef et al., 2017).

### DNA Extraction

The amniotic fluid and peripheral blood samples were kept at −20°C until DNA isolation. DNA extraction was performed using the QIAamp DNA Blood Mini Kit (Qiagen, Duesseldorf, Germany), according to the manufacturer's protocol. The NanoDrop 2000 (Thermo Fisher Scientific, USA) was used to estimate isolated DNA concentrations.

### MLPA

MLPA was performed using the SALSA MLPA P034 and P035 kits (MRCHolland, Amsterdam, the Netherlands) on an ABI Prism 3100 Genetic Analyzer (Applied Biosystems, CA, USA) with Coffalyser Net software (http://www.coffalyser.net).

### Gap-PCR

To determine the exact breakpoints of the deletion involving the exon 51, specific primer sets were designed on both sides of the breakpoints. The reaction was performed on TaKaRa LA Taq^TM^ (TaKaRa Bio Inc.) as previously described (Qian et al., 2019). The procedure of the PCR was as following: 94°C for 90 s, then followed by 12 cycles of 94°C for 30 s, 72°C for 20 s (−1°C per cycle), 72°C for 45 s, then another 35 cycles of 94°C for 30 s, 60°C for 20 s, 72°C for 45 s, and a final extension at 72°C for 5 min. Finally, the following primer sets (forward primer: 5′-ACCACACGGAACTTAAAGGATTGA-3′; reverse primer: 5′-ACCTGGGATCTAGTCCTCATTTG-3′) were determined.

### Droplet Digital PCR

The mutations were detected using the Droplet Digital PCR system [Pilot Gene Technologies (Hangzhou) company ltd, China]. Samples were prepared as previously described (Qian et al., 2019). The primers and probes were designed to detect the deletions in the wild allele and the mutant allele (Table 1). Thermal cycling started with a denaturation step of 95°C for 10 min, then, followed by 40 cycles of 95°C for 30 s, 55°C for 30 s, and 72°C for 30 s.

**Table 1:** The primers and probes for ddPCR.

**Primer**	**Sequence (5^**′**^-3^**′**^)**	**Product length (bp)**
DMDdel-F	CAGATTCGCAGGCTTTTGATATT	23
DMDdel-R	CACTGAAAACTAAGTTTTTGATAGCTTACG	30
DMDdel-P	FAM- TGGCCCAGTAACATAAG–MGB	17
DMDwt-F	CCATCAATAAACTGAGGCAAAGC	23
DMDwt-R	CGCCTCTAGATAATCACTCATTTCTTC	27
DMDwt-P	VIC-CCATTCGCTGATACTACCT -MGB	19

## Results

### Recurrent Occurrence of the Exon 51 Deletion

**Figure 1:**
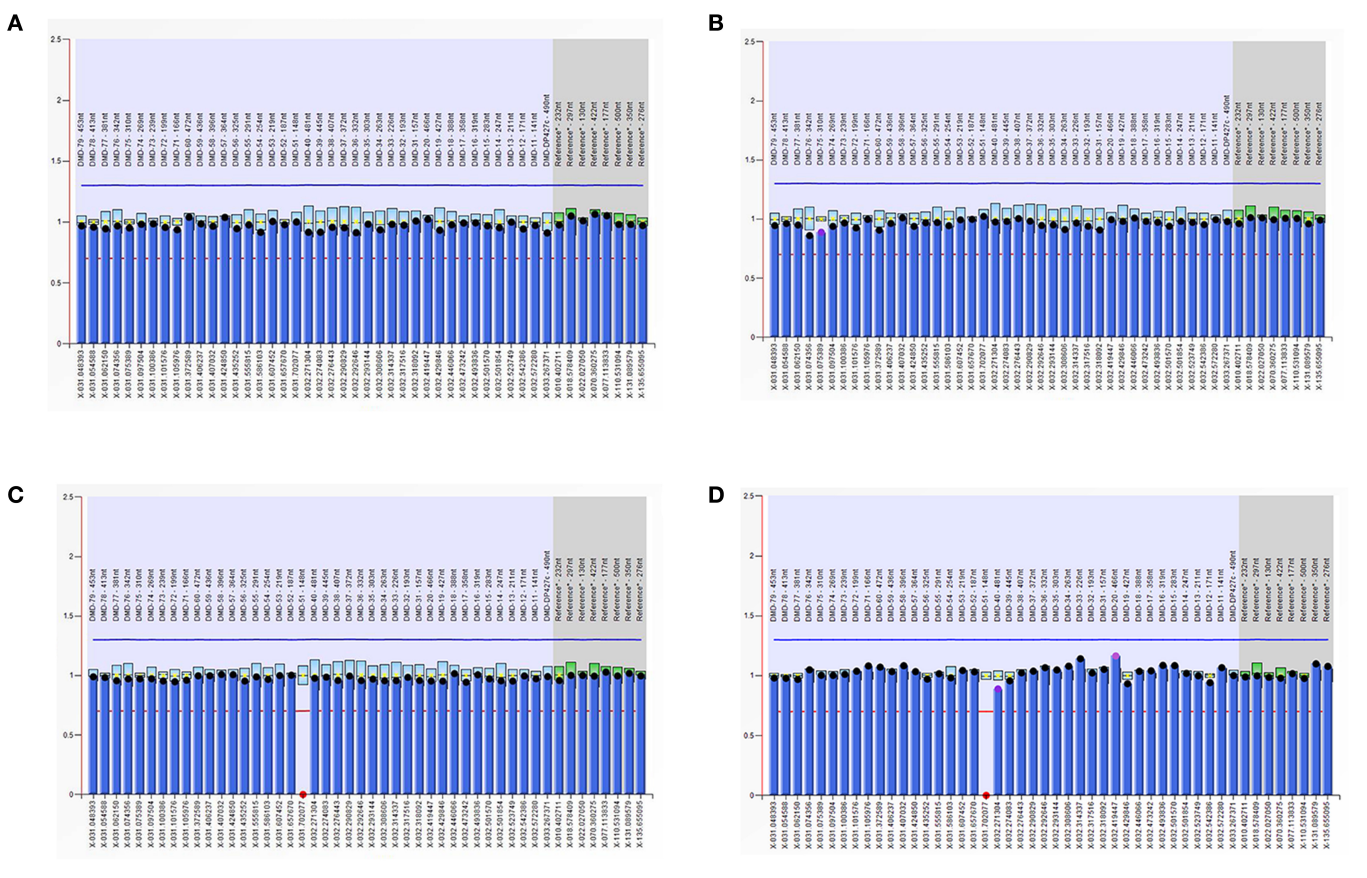
Multiplex ligand-dependent probe amplification (MLPA) results. The ordinate represents the gene copy number, the abscissa represents the chromosomal position of the gene. The red dots in the MLPA chart indicate the deletion of the exon 51 in the dystrophin gene in the proband and the fetus, respectively. **(A)** DNA from the proband's father; **(B)** DNA from the proband's mother; **(C)** DNA from the proband; **(D)** DNA from the fetus.

**Figure 2:**
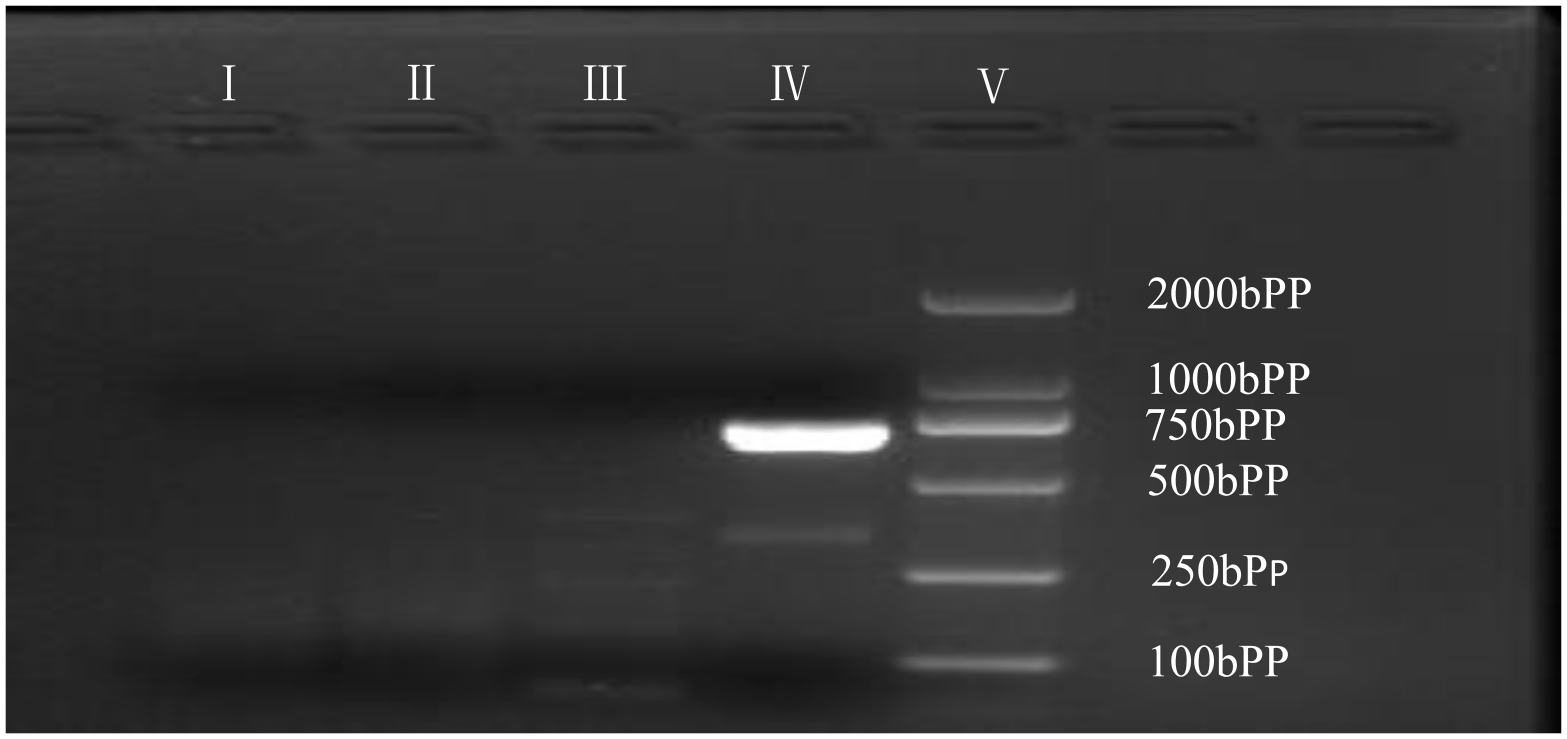
Gap-PCR of family members. Gap-PCR was performed to amplify a putative breakpoint junction sequence. Lane I–V: blank contrast, father, mother, proband, marker. The 750 bp band corresponding to the deletion mutation is present in line IV.

**Figure 3:**
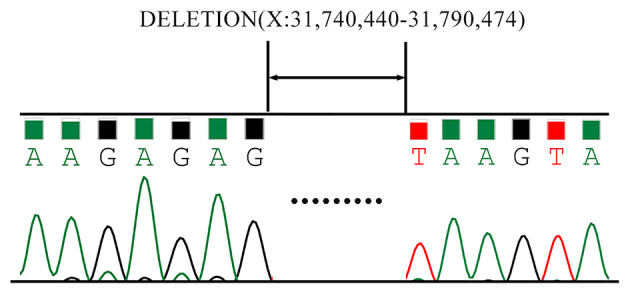
Sanger Sequencing results of the breakpoint junction sequence. The deletion spans 50,035 bp, locating in X: 31,740,440-31,790,474. It contains the whole exon 51, part of the intron 50 and the intron 51.

As is Figure 1, a deletion in the exon 51 was detected both in the proband and the amniotic fluid. Then, specific primer sets were designed and gap-PCR was performed to amplify the breakpoint junction sequence. According to the gel electrophoresis results (Figure 2), the band corresponding to the breakpoint junction sequence presents only in the proband, about 750 bp in size. Sanger sequencing and blast searching in NCBI show that the deletion spans 50,035 bp, locating in X: 31,740,440-31,790,474. It contains the whole exon 51, part of the intron 50 and the intron 51 (Figure 3).

### Determination of the Maternal Mosaicism

**Figure 4:**
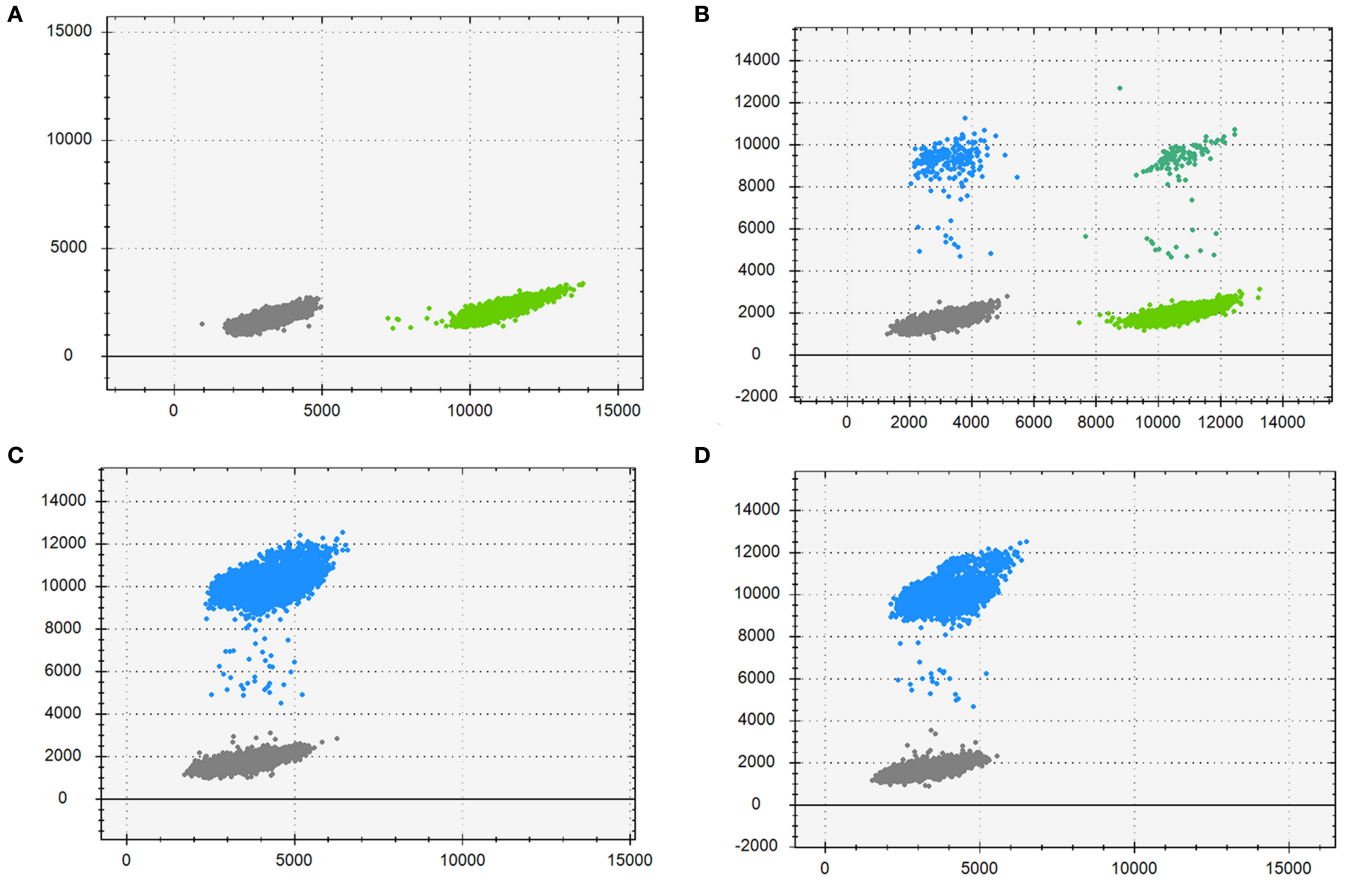
Droplet digital PCR results for the mutation mosaicism analysis in the pedigree. Blue dots, droplets contain FAM-labeled fluorescence probes only, detecting the mutant allele; Cyan dots, droplets contain VIC-labeled fluorescence probes only, detecting the wild allele; Gray dots, droplets without neither fluorescence probes; Green dots, droplets with both fluorescence probes. **(A)** DNA from the proband's father; **(B)** DNA from the proband's mother; **(C)** DNA from the proband; **(D)** DNA from the fetus.

DdPCR was performed to estimate the mutation frequency. As is Figure 4, the mother (Figure 4B) had a low-level pathogenic mutant allele dots as her morbid children did (Figures 4C,D). Further study identified that the mother had a mutation frequency of 3.53% (Table 2). Therefore, the maternal origin for the deletion mutation was clear.

**Table 2:** Mutation frequencies in the pedigree.

**Subject**	**Number**	**Mutation concentration (copies/μl)**	**Wild type concentration (copies/μl)**	**Mutant frequency % (on average)**
Proband	NO.1	1077.14	0	100
	NO.2	1072.63	0	
Fetus	NO.1	932.05	0	100
	NO.2	927.96	0	
Father	NO.1	0	975.04	0
	NO.2	0	976.42	
Mother	NO.1	30.17	862.03	3.53%
	NO.2	29.76	833.49	

*The samples were collected from the amniotic fluid in the fetus and peripheral blood in the other family members. Mutant frequencies were calculated based on the average of two samples*.

## Discussion

In this pedigree, prenatal diagnosis indicated that the fetus had the same deletion in the exon 51 of the dystrophin gene as that in the proband. In addition, a low-level somatic mutation mosaicism in the exon 51 of the dystrophin gene was identified in the healthy mother. Considering that DMD is a X-linked genetic disease, the genotyping in the fetus is explicit–the fetus is male and the mutation is pathogenic. Though some treatments such as deflazacort were approved by the United States Food and Drug Administration (FDA) to treat DMD, the treatments currently available were palliative (Falzarano et al., 2015; Venugopal and Pavlakis, 2021). Finally, the couple decided to terminate the pregnancy at 24 weeks of gestation, after receiving the genetic consultation and careful consideration.

DMD is a neuromuscular disorder caused by mutations in the dystrophin gene, affecting 1 in 3,500 males (Wang et al., 2017; Fox et al., 2020). The major symptoms are motor disability or gait abnormality. Less frequent presentations include: dilated cardiomyopathy, intellectual disability, chronic respiratory dysfunction, and scoliosis (Yiu and Kornberg, 2015; Tsuda, 2018).

The dystrophin gene, composed of 79 exons, is one of the largest genes identified in human beings. Mutations in the dystrophin gene are proved to be the primary cause for DMD, including deletions (57–70%), duplications (5–10%), and point mutations (30–35%) (Robinson-Hamm and Gersbach, 2016; Yang et al., 2019). Approximately, one third of the identified mutations are inherited from asymptomatic parents, which are defined as DNMs (Haldane, 2004; Wilfert et al., 2017). However, among all the DNMs pedigrees, the incidences of germline mosaicism are reportedly varied from 3.3 to 20% (Grimm et al., 2012; Qin et al., 2016; Dai et al., 2020).

Germline mosaicism in DMD was first observed in a Netherlands' family in 1987 (Bakker et al., 1987). Patel et al. reported that the detection of low-level somatic mosaicism with germline components was an available way to identify germline mosaicism (Patel et al., 2018). Low-level mosaicism means a mutation percentage of <10% in allele fractions (AFs) (Karolak et al., 2020). To detect different types of mutations, specific techniques are applied, generally, next generation sequencing (NGS), MLPA and ddPCR.

It is reported that NGS is capable to detect AFs as low as 1% (Brewer et al., 2020). In general, the sequencing capability depends on the depth of sequencing. Briefly, 200X depth coverage can reveal a mutation frequency of 3% and 500X depth coverage is believed to be stable enough for low-level mutation mosaicism detection (Dai et al., 2020; Lin et al., 2021). Comparing with routine whole-exon sequencing or whole-genome sequencing, which is 30-100X depth coverage on average, the depth coverage for low-level mutation mosaicism detection with NGS is costly (Postel et al., 2018).

MLPA is clinically a preferred method for the detection of a deletion mutation (Luce et al., 2014). The evaluation of copy number is determined according to the intensity ratio of sample to control. Briefly, deletions are considered when the ratio is <0.65, and duplications are recognized if the ratio is >1.30. While the relative signal strength in MLPA is mainly affected by the copy number of the probe target sequence, and till now, the detectable threshold of mosaicism percentage remains ambiguous. Therefore, MLPA is not recommended for the routine identification of low-level mosaicism (Schouten et al., 2019).

Digital PCR (dPCR) is an absolute quantitative method with greater precision and improved reproducibility compared with real-time PCR (Hindson et al., 2013). The target DNA samples are distributed into multi-well plates and PCR is performed respectively. As a result of the isolated analysis of reactions in each well after limiting dilution, the relative concentration of the target DNA is high, greatly improving its sensitivity and precision (Hindson et al., 2011). DdPCR is an advanced development based on the traditional digital PCR. Instead of the multi-well plates, the reaction mixture is divided into nanoliter-sized droplets in oil. Such vast numbers of aqueous droplets greatly improve the sensitivity and accuracy of the dPCR. Therefore, the concentrations of the target DNA can be detected even in a low-level dilution (Pinheiro et al., 2012).

To our knowledge, ddPCR has been used to detect deletion/duplication mutation mosaicism as well as point mutation mosaicism in previous studies (Qian et al., 2019; Jin et al., 2020). Our study is another verification of it. Nowadays, ddPCR has been used in many areas where require precise identification. For example, it has an application of monitoring of the therapeutic effectiveness of exon skip-inducing antisense oligonucleotides in clinical studies (Verheul et al., 2016).

It was reported that the deletions preferentially occurred in oogenesis (Grimm et al., 1994, 2012), meaning that the identification of female carriers was important. Apart from the genetic detection technologies mentioned above, some biomarkers such as the creatine phosphokinase (CPK), hemopexin (Danieli and Angelini, 1976), myostatin (GDF-8) (Anaya-Segura et al., 2015), MiR-133b and miR-499 (Zhang et al., 2020) were reportedly suitable for carriers detection. However, for those low-level mutation mosaic carriers, especially the ones who had no clinical manifestations, the biomarkers above might be incapable (Anaya-Segura et al., 2015). Therefore, for those asymptomatic couples who suffered recurrent occurrence of the DNMs, ddPCR might be a promising technology.

In conclusion, this is the first report of a pedigree with low-level somatic mosaicism caused by the exon 51 deletion detected by ddPCR. It is of great significance for the genetic counseling for this affected pedigree. It is also a warning sign that cautions should be taken when estimating recurrent risks of DNMs, especially the repeated occurrence of DNMs in more than two offspring with healthy parents.

## Data Availability Statement

The datasets presented in this study can be found in online repositories. The names of the repository/repositories and accession number(s) can be found in the article/supplementary material.

## Ethics Statement

The studies involving human participants were reviewed and approved by the Ethics Committee of Women's Hospital, School of Medicine Zhejiang University. Written informed consent to participate in this study was provided by the participants' legal guardian/next of kin. Written informed consent was obtained from the individual(s), and minor(s)' legal guardian/next of kin, for the publication of any potentially identifiable images or data included in this article.

## Author Contributions

MD conceived of the study. MD and PJ participated in its design and PJ drafted the manuscript. YaX and YY collected the samples and clinical data. MW, YQ, XG, and JY carried out the gap-PCR, MLPA, and ddPCR. YuX helped to revise the manuscript. All authors have read and approved the final manuscript.

## Conflict of Interest

The authors declare that the research was conducted in the absence of any commercial or financial relationships that could be construed as a potential conflict of interest.
